# Inhibition of the NFATc4/ERK/AKT Pathway and Improvement of Thiol-Specific Oxidative Stress by Dronedarone Possibly Secondary to the Reduction of Blood Pressure in an Animal Model of Ventricular Hypertrophy

**DOI:** 10.3389/fphys.2020.00967

**Published:** 2020-08-26

**Authors:** Laia Pazó-Sayós, Maria Carmen González, Begoña Quintana-Villamandos

**Affiliations:** ^1^Department of Anesthesiology and Intensive Care, Hospital Gregorio Marañón, Madrid, Spain; ^2^Department of Physiology, Faculty of Medicine, Universidad Autónoma, Madrid, Spain; ^3^Department of Pharmacology and Toxicology, Faculty of Medicine, Universidad Complutense, Madrid, Spain

**Keywords:** dronedarone, hypertension, left ventricular hypertrophy, oxidative stress, protein thiolation index

## Abstract

Untreated chronic hypertension causes left ventricular hypertrophy, which is related to the occurrence of atrial fibrillation. Dronedarone is an antiarrhythmic agent recently approved for atrial fibrillation. Our group previously demonstrated that dronedarone produced an early regression of left ventricular hypertrophy after 14 days of treatment in an experimental study. In this study, we analyze the possible mechanisms responsible for this effect. Ten-month-old male spontaneously hypertensive rats (SHRs, *n* = 16) were randomly divided into therapy groups: SHR-D, which received dronedarone, and hypertensive controls, SHR, which received saline. Ten-month-old male Wistar Kyoto rats (WKY, *n* = 8), which also received a saline solution, were selected as normotensive controls. After 14 days of treatment, echocardiographic measurements of the left ventricle were performed, blood samples were collected for thiol-specific oxidative stress analysis, and the left ventricles were processed for western blot analysis. Dronedarone significantly lowered the left ventricular mass index and relative wall thickness compared with the SHR control group, and no differences were observed between the SHR-D group and the WKY rats. Interestingly, the SHR-D group showed significantly decreased levels of nuclear factor of activated T cells 4 (p-NFATc4), extracellular-signal-regulated kinase 1/2 (p-ERK1/2), and protein kinase B (p-AKT) compared with the hypertensive controls without statistical differences when compared with the WKY rats. Moreover, the SHR control group showed elevated thiolated protein levels and protein thiolation index (PTI) compared with the WKY rats. After treatment with dronedarone, both parameters decreased with respect to the SHR control group until reaching similar levels to the WKY rats. Our study suggests that dronedarone produces inhibition of the NFATc4/ERK/AKT pathway and improvement of thiol-specific oxidative stress possibly secondary to the reduction of blood pressure in an animal model of ventricular hypertrophy.

## Introduction

Hypertension is a very common disease among the general population (Banegas and Gijón-Conde, 2017). Untreated chronic high blood pressure levels result in left ventricular hypertrophy (LVH) that in time may progress to unstable cardiomyopathy (Burchfield et al., 2013). This increases heart fibrosis due to pressure overload, affects the correct distribution of electric signals among cardiomyocytes, and may trigger atrial fibrillation (AF; Yildiz et al., 2020).

There have been several clinical and preclinical studies that link the chronic use of antihypertensive therapy to a regression in LVH (Degirmenci et al., 2014; Salvetti et al., 2018; Jekell et al., 2018). Our group previously demonstrated that esmolol, an endovenous and selective beta-blocking agent, produces early regression in LVH and vascular remodeling after 48 h of treatment in an experimental model of arterial hypertension (Quintana-Villamandos et al., 2013, 2016a,b) *via* inhibition of AKT/NF-𝜅B and NFATc4 and reduction of oxidative stress (Quintana-Villamandos et al., 2018).

Numerous signaling pathways have been associated with the development of cardiac hypertrophy. Proteins such as NFATc4, ERK1/2, AKT, or NF-κB have been related to hypertrophy in different animal assays (Bernardo et al., 2010; van Berlo et al., 2013; Schirone et al., 2017). As previously mentioned (Quintana-Villamandos et al., 2018), drugs that interfere with these signaling cascades have demonstrated to reduce or prevent the development of this hypertrophy (Zhu et al., 2015; Ba et al., 2019). On the other hand, an enhanced oxidative stress status may be related to the induction or maintenance of cardiac remodeling by its action on these signaling pathways (Maulik and Kumar, 2012; Sag et al., 2014; Moris et al., 2017). Recently, our group has demonstrated that patients with LVH show increased levels of protein thiolation index (PTI; Quintana-Villamandos et al., 2019).

Dronedarone is a novel antiarrhythmic agent derived from amiodarone that has recently been approved for AF treatment (Camm et al., 2012; January et al., 2014). Though its mechanism of action is not fully understood, we do know that dronedarone acts as a blocking agent in calcium, sodium, and potassium channels and it exerts anti-adrenergic properties. Dronedarone reduces AF recurrence after cardioversion, whether paroxysmal or persistent, and cardiovascular-related hospitalizations and deaths (Singh et al., 2007; Connolly et al., 2009). However, it may worsen the clinical status of hemodynamically unstable patients or those with moderate to severe ventricular dysfunction (Kober et al., 2008). Our group previously demonstrated that dronedarone produced regression of LVH after only 14 days of treatment in a preclinical study (Quintana-Villamandos et al., 2017). In the present study, we aim to analyze the possible mechanisms responsible for this effect.

## Materials and Methods

All procedures fulfilled the stipulations of the *Guidelines for the Care and Use of Laboratory Animals* (Directive 2010/63/EU and Spanish Law RD 53/2013) and were approved by the Ethics Committee of Hospital General Universitario Gregorio Marañón, Madrid, Spain.

### Experimental Design

The study animals, 10-month-old spontaneously hypertensive rats (SHRs; *n* = 16), and WKY (*n* = 8), as normotensive controls, were bred at the animal facility of Universidad Autónoma de Madrid. As described in detail in previous studies (Quintana-Villamandos et al., 2017), all rats were supplied with standard rat chow and drinking water *ad libitum* and were maintained on a 12/12 h light/dark cycle. The animals were housed at a constant temperature of 24°C and relative humidity of 40%. The SHRs were randomly divided into two groups: rats that received oral treatment with dronedarone (SHR-D, *n* = 8) and a hypertensive control group (SHR, *n* = 8) treated with a vehicle. The SHR-D group was treated with dronedarone 100 mg/kg once daily for 14 days and administered and dissolved in saline by intragastric tube. The WKY and SHR control groups received a saline solution as a vehicle during the same period of time. When treatment was completed, an echocardiographic study of the left ventricle was performed after sedation with an intraperitoneal injection of diazepam 4 mg/kg and ketamine 10 mg/kg, and blood samples were collected for thiol-specific oxidative stress analysis. Finally, the rats were euthanized by decapitation, the hearts were collected immediately, and left ventricles were bisected and processed for western blot analysis.

### Blood Pressure and Heart Rate Measurements

Conscious animals were pre-warmed at 35°C in thermostatic cages. Afterward, their systolic blood pressure (SBP) and heart rate (HR) were measured by the tail-cuff method with a photoelectric sensor (Niprem 546, Cibertec, Madrid, Spain).

### Echocardiographic Study

As described in detail previously (Quintana-Villamandos et al., 2017), transthoracic echocardiography was performed using the VIVID q system (GE Healthcare, Germany) equipped with a 13-MHz probe (12S-RS, GE). Transthoracic echocardiography was performed under anesthesia after 14 days of treatment. M-mode imaging of the parasternal short axis (papillary level) enabled the measurement of the left ventricular internal diastolic diameter (LVIDd), the posterior wall diastolic thickness (PWd), and the interventricular septal end-diastolic thickness (IVSd). The left ventricular mass (LVM) was adjusted for body weight [left ventricular mass index (LVMI)] and relative wall thickness (RWT), were calculated as previously described (Quintana-Villamandos et al., 2013):

$$\begin{array}{l} \mathrm{LVM}=0.8\;[1.04\;{\left(\mathrm{IVSd}+\mathrm{LVIDd}+\mathrm{PWd}\right)}^{3}\\ \;\;\;\;\;\;\;\;\;\;\;\;\;\;\;-{\left(\mathrm{LVIDd}\right)}^{3}]+0.6\;g \end{array}$$

$$\mathrm{RWT}=\left(\mathrm{PWd}+\mathrm{IVSd}\right)/\mathrm{LVIDd}$$

### Sample Preparation

The same protocols described in previous studies (Quintana-Villamandos et al., 2018) were followed in the present one. We homogenized the left ventricular tissue samples using a TissueLyser LT system (QIAGEN, Hilder, Germany) programmed with 50 s^−1^ oscillation for 4 min in a lysis buffer containing 20 mM Tris-HCl buffer (pH 7.5), 150 mM NaCl, 1 mM EDTA, 1% Triton X-100, 20 mM sodium orthovanadate, 1 mM sodium fluoride, 1 mM PMSF, and 1% protein inhibitor cocktail acquired from Sigma-Aldrich (Madrid, Spain). Homogenates were centrifuged at 10,000 *g* for 2 min at 4°C and the supernatant was stored at −80°C until analysis.

Blood samples were centrifuged at 900 *g* and 4°C for 10 min. The plasma obtained was stored at −80°C until analysis.

### Western Blot Analysis

As described in detail previously (Quintana-Villamandos et al., 2018), we used an immunoblotting technique to analyze the total (T-) and phosphorylated (p-) levels of NFATc4, AKT, ERK, and NF-κB in the left ventricular homogenates. Electrophoresis was performed with the Mini-Protean Tetra system (Bio-Rad, Madrid, Spain) at 100 V and room temperature. SDS-PAGE electrophoresis on 12% gel was used to separate a 40 μg protein sample, which was transferred onto an Immunoblot polyvinylidene difluoride (PVDF) membrane (Bio-Rad, Madrid, Spain) for 1 h at 120 V and 4°C. We used a stain kit (MemCode, Thermo Scientific, Madrid, Spain) and reversible protein to check the efficiency of the protein transfer. Cell Signaling Technology (MA, USA) provided us with primary antibodies against total (T) and phosphorylated (p-) NFATc4 [Santa Cruz Biotechnology, Germany; dilution factor (DF) = 1:200, 140 KDa], p-ERK (DF = 1:2000, 42 KDa), p-AKT (DF = 1:1000, 60 KDa), and p-NF-𝜅B (DF = 1:1000, 65 KDa). After overnight incubation of primary antibodies at 4°C and previous washing with 1xPBS, the resultant PVDF membrane was incubated again overnight at room temperature with IgG-peroxidase-conjugated secondary anti-rabbit (DF = 1:2000) and anti-mouse (DF = 1:2000) antibodies (Cell Signaling Technology, USA). We performed another washing and incubated blots with the SuperSignal West Pico Chemiluminescent substrate kit (Thermo Scientific, Madrid, Spain). Using a gel documentation and analysis system (Alliance, Uvitec, Cambridge, UK), protein expression bands were obtained. Using the free ImageJ NIH software application, we analyzed the density of the bands on the film. To correct for protein loading, we normalized the values using a GAPDH antibody (Millipore, Madrid, Spain; DF = 1:2000).

### Oxidative Stress Biomarkers

#### Total Thiols

Total thiol levels in the plasma were determined using Ellman’s reagent [5,5'-dithio-bis (2-nitrobenzoic acid) or DTNB] adapted to nanovolume. Thiol groups, present in the proteins or the low molecular weight compounds, react with the DTNB, which is reduced to thiol 5-thionitrobenzoic acid (TNB), and is yellow in color, and can be measured and quantified at 412 nm in a Nanodrop 2000 spectrophotometer (Thermo Scientific, NC, USA).

#### Thiolated Proteins

To analyze the concentration of thiolated proteins, the method described by Giustarini et al. (2012) was used (Colombo et al., 2015), which is based on the detection of thiolated proteins by means of a spectrophotometry method by binding to the ninhydrin reagent. The ninhydrin reagent emits at a 570 nm wavelength, which can be measured and quantified in a Nanodrop 2000 spectrophotometer (Thermo Scientific, NC, USA).

#### Protein Content

To determine the protein content, the Bradford method was used, following the protocol suggested by the manufacturer. After 5 min of sample reaction with the Bradford reagent (Coomassie blue dye, Bio-Rad, Spain), absorbance at 595 nm was measured in a plate reader (Synergy HT Multi-Mode Microplate Reader, Biotek, Rochester, VT, USA). Protein concentration (μg/μl) in the sample was estimated from the calibration line with increasing concentrations (range 0.1–0.5 μg/μl) of bovine serum albumin.

#### Protein Thiolation Index

PTI was calculated as the molar ratio between S-thiolated proteins and the concentration of free, DTNB-titratable protein-SH group (total thiols; Giustarini et al., 2012; Colombo et al., 2015).

### Statistical Analysis

All results were expressed as mean ± SEM. The parameters were compared by a single-factor (rat) analysis of variance (ANOVA). A *post hoc* Bonferroni correction was applied. Statistical significance was set at *p* ≤ 0.05. To perform the analysis, IBM SPSS Statistics for Windows, version 20.0 (IBM Corp, Armonk, New York, USA) and Prism GraphPad 6.0 (GraphPad Software, California, USA) were used.

## Results

### Dronedarone Reduces Heart Rate and Blood Pressure

Table 1 shows the values of the physiological parameters. The SHR and WKY groups showed similar HR values, whereas the SHR-D group displayed a significant reduction of this parameter (*p* < 0.001). The SHR control group had a markedly higher SBP compared with the WKY rats (*p* < 0.001). Oral administration of dronedarone for 14 days resulted in a significantly lower SBP compared with the SHR control group (*p* < 0.001) No statistical differences in SBP were shown between the WKY rats and the SHR-D group.

**Table 1:** Weight, heart rate, and systolic blood pressure from all groups.

	WKY (*n* = 8)	SHR (*n* = 8)	SHR-D (*n* = 8)
Body weight (g)	460.67 ± 2.6	383.33 ± 3.9^***^	375.67 ± 2.8^***^
SBP (mmHg)	136 ± 2	174 ± 2^***^	142 ± 2^###^
HR (bpm)	392 ± 3	392 ± 3	314 ± 5^***^,^###^

SBP, systolic blood pressure; HR, heart rate; WKY, Wistar-Kyoto rats; SHR, spontaneously hypertensive rats; SHR-D, spontaneously hypertensive rats treated with dronedarone. Statistically significant differences between WKY, SHR, and SHR-D are shown as follows:

*** *p* < 0.001 vs. WKY; and

### *p* < 0.001 vs. SHR. Values are shown as mean ± SEM.

### Dronedarone Produces Left Ventricular Geometry Changes

**Figure 1:**
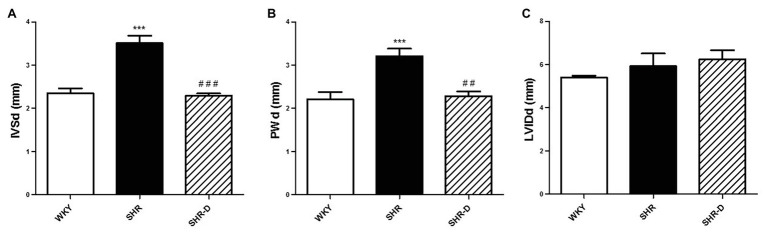
Echocardiographic parameters with M-mode measurements. **(A)** Interventricular septal end-diastolic thickness (IVSd), **(B)** posterior wall diastolic thickness (PWd), and **(C)** left ventricular internal diastolic diameter (LVIDd) measurements from Wistar-Kyoto rats (WKY) treated with vehicle, spontaneously hypertensive rats (SHRs) treated with vehicle, and spontaneously hypertensive rats treated with dronedarone (SHR-D). Statistically significant differences among WKY, SHR, and SHR-D are shown (^***^p < 0.001 vs. WKY; ^##^*p* < 0.01 vs. SHR; and ^###^*p* < 0.001 vs. SHR). Values are shown as mean ± SEM (*n* = 8 per group).

**Figure 2:**
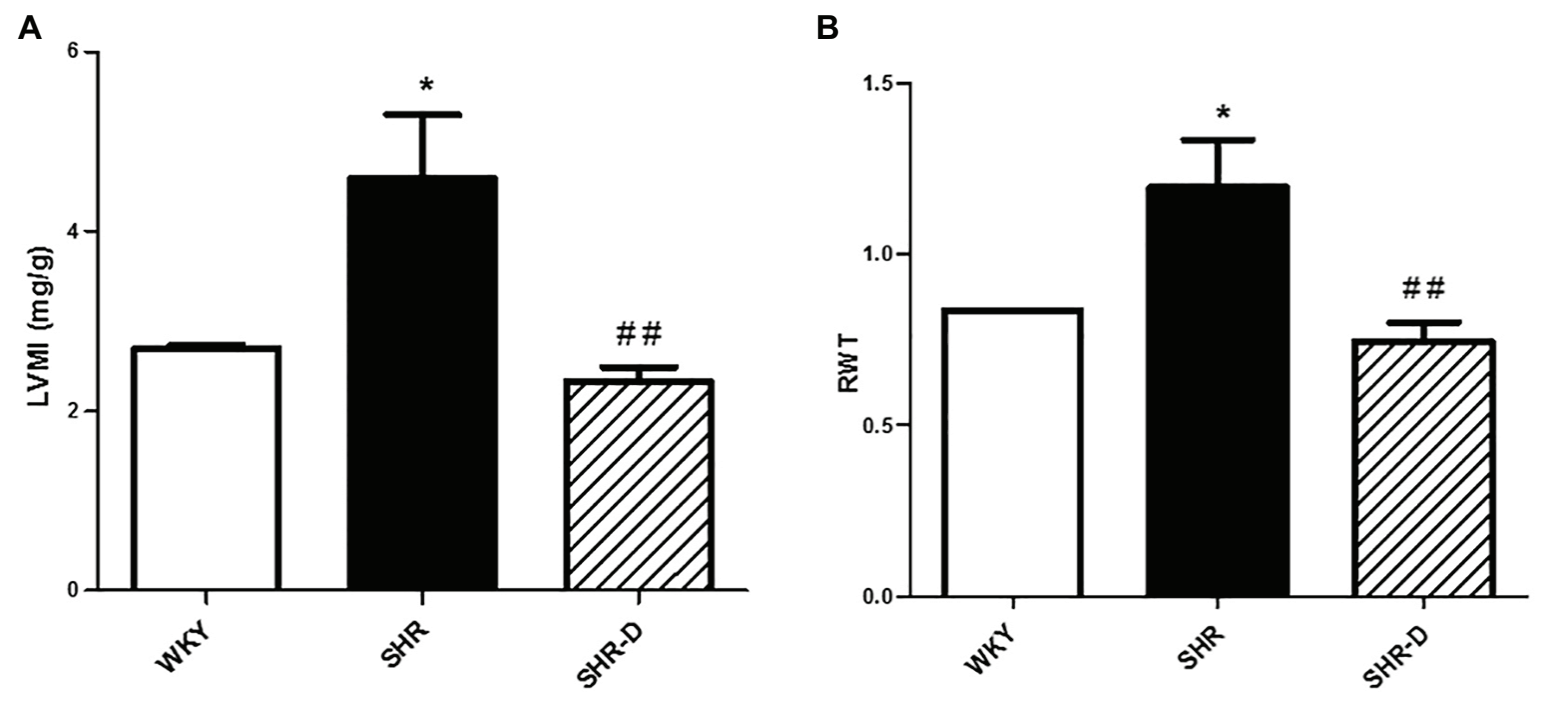
Parameters indicative of left ventricular geometry. **(A)** Left ventricular mass index (LVMI) and **(B)** relative wall thickness (RWT) measurements from Wistar-Kyoto rats (WKY) treated with vehicle, spontaneously hypertensive rats (SHR) treated with vehicle, and spontaneously hypertensive rats treated with dronedarone (SHR-D). Statistically significant differences among WKY, SHR, and SHR-D are shown (^*^p < 0.05 vs. WKY; and ^##^*p* < 0.01 vs. SHR). Values are shown as mean ± SEM (*n* = 8 per group).

Figures 1, 2 show parameters indicative of left ventricular geometry. IVSd and PWd of the SHR control group in comparison with the WKY rats increased (*p* < 0.001 and *p* < 0.001, respectively). Two weeks of dronedarone administration decreased IVSd (*p* < 0.001) and PWd (*p* < 0.01) in the SHR-D group, and no differences were detected with respect to the WKY rats (Figures 1A,B). The SHR control group shows concentric LVH (increased LVMI and RWT) when compared with the WKY rats (LVMI *p* < 0.05; RWT *p* < 0.05). Oral administration of dronedarone resulted in a lower LVMI and RWT compared with the SHR control group (LVMI *p* < 0.01; RWT *p* < 0.01). The SHR-D group showed normal left ventricular geometry because no statistical differences in either LVMI or RWT were shown with respect to the WKY rats (Figures 2A,B).

### Dronedarone Improves the Cellular Signalization: NFATc4/ERK/AKT Pathway

**Figure 3:**
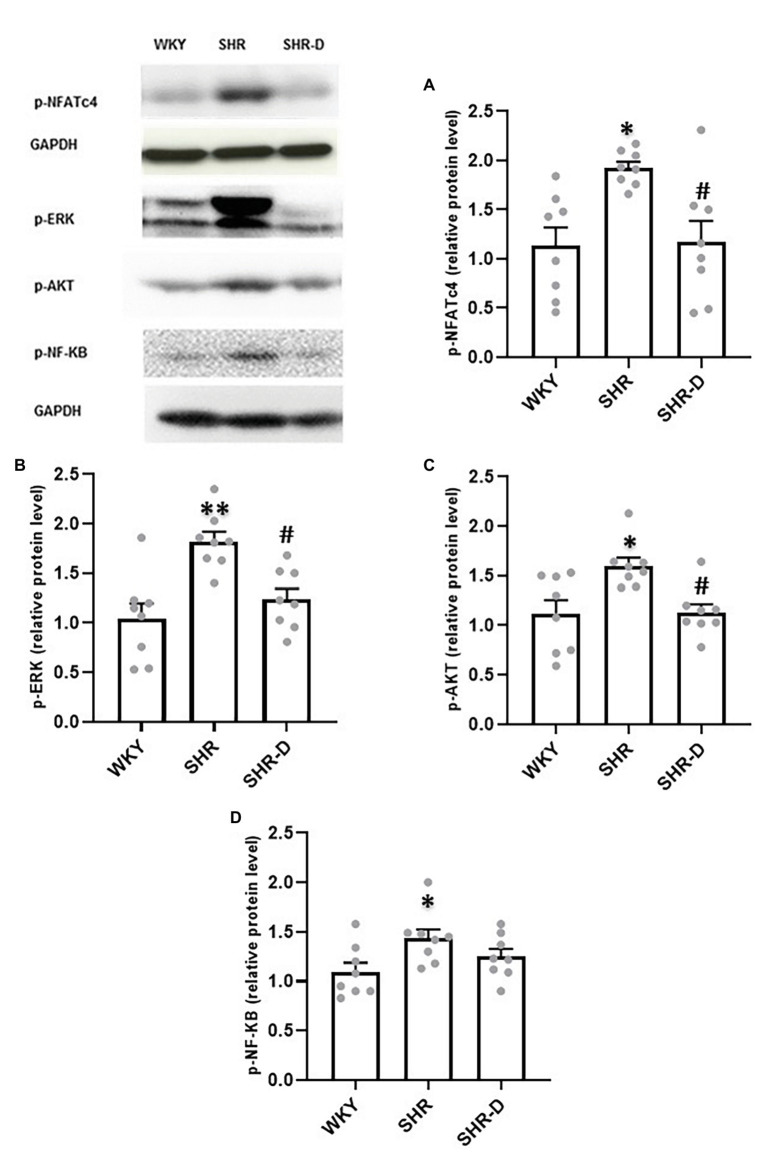
Western blot analysis in the left ventricular myocardium. Representation of **(A)** p-NFATc4, **(B)** p-ERK1/2, **(C)** p-AKT, and **(D)** p-NF-κB levels from WKY treated with vehicle, SHR treated with vehicle, and SHR-D. Statistically significant differences among WKY, SHR, and SHR-D are shown (^*^p < 0.05 vs. WKY; ^**^p < 0.01 vs. WKY; and ^#^*p* < 0.05 vs. SHR). Values are shown as mean ± SEM (*n* = 8 per group).

Figure 3 shows the effects of dronedarone on the different signaling cascades. p-NFATc4 (Figure 3A) and ERK (Figure 3B) levels present in the left ventricular myocardial homogenates of the SHR control group were significantly higher than in the WKY controls (*p* < 0.05 and *p* < 0.01, respectively). After treatment with dronedarone, these levels decreased compared with the hypertensive controls (*p* < 0.05). No differences were observed between the WKY and SHR-D rats.

Similarly, and as previously stated (Quintana-Villamandos et al., 2018), phosphorylated AKT levels are higher in the SHR control group compared with the WKY rats (*p* < 0.05). However, as shown in Figure 3C, dronedarone produced a significant decrease in p-AKT levels compared with the hypertensive control group (*p* < 0.05). Interestingly, once again, no statistical differences were found between the SHR-D and WKY groups.

On the other hand, although p-NF-κB expression was enhanced in the SHR control group (Figure 3D), dronedarone did not produce significant change in its expression.

### Dronedarone Improves Thiol-Specific Oxidative Stress

**Figure 4:**
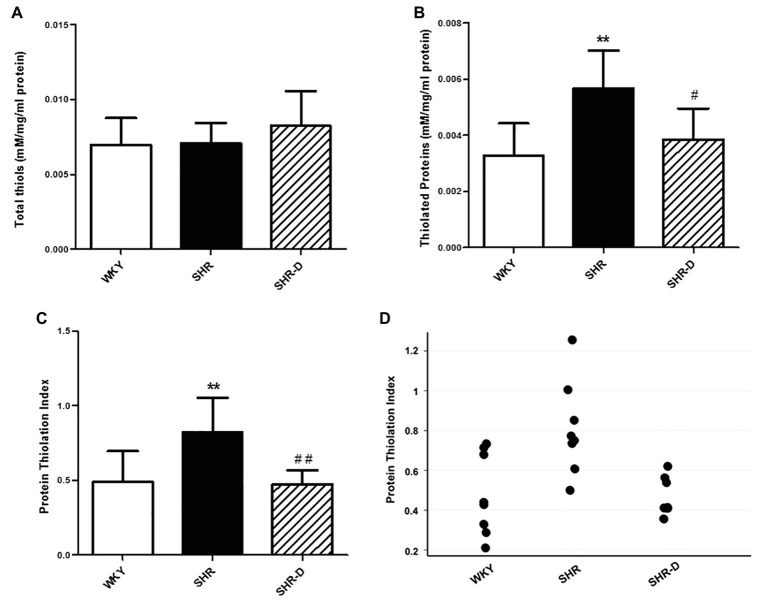
Thiol-specific oxidative stress analysis. **(A)** Plasma concentration of thiols, **(B)** thiolated proteins, **(C)** protein thiolation index (PTI), and **(D)** data points of the protein thiolation index from WKY treated with vehicle, SHR treated with vehicle, and SHR-D treated with dronedarone. Statistically significant differences among WKY, SHR, and SHR-D are shown (^**^p < 0.01 vs. WKY; ^#^*p* < 0.05 vs. SHR; and ^##^*p* < 0.01 vs. SHR). Values are shown as mean ± SEM (*n* = 8 per group).

Figure 4 shows the results of the thiol-specific oxidative stress analysis. No significant differences were observed in the thiol plasma concentration among the three groups (Figure 4A), but there was a higher concentration of thiolated proteins in the SHR group with respect to the WKY group (*p* < 0.01; Figure 4B). After treatment with dronedarone, a decrease in the concentration of thiolated proteins was observed in the SHR-D group with respect to the SHR control group (*p* < 0.05; Figure 4B). When calculating PTI, the SHR control group showed a markedly higher PTI than the WKY control group (*p* < 0.01). Treatment with dronedarone produced a decrease of PTI (*p* < 0.01) to match the group of WKY control rats (Figures 4C,D).

## Discussion

Our results suggest that dronedarone produces inhibition of the NFATc4/ERK/AKT pathway and improvement of thiol-specific oxidative stress possibly secondary to the reduction of blood pressure.

The SHR animal model has been used widely as an experimental model for LVH (Quintana-Villamandos et al., 2013; Zimmer et al., 2015; Lu et al., 2016; Zwadlo and Borlak, 2016; Salvetti et al., 2018). Ten-month-old male rats show a hypertension-related compensated LVH (Brooks et al., 2009). Our group previously demonstrated that short-term (14 days) treatment with dronedarone (100 mg/kg orally and once daily) produced LVH regression (dronedarone reduced LVM; this finding coincided with changes in the cardiomyocytes, collagen, and myocardial glucose metabolism of the left ventricle; Quintana-Villamandos et al., 2017). However, the mechanisms involved in this regression are unknown to date.

### Effects of Dronedarone on HR and SBP

Dronedarone acts as a blocking agent on multiple ion currents. It is considered to be a class III antiarrhythmic drug, but, like amiodarone, it exhibits effects attributable to all four Vaughan-Williams classes (sodium channel blockers, beta and alpha-blockers, potassium channel blockers, and non-dihydropyridine calcium channel blockers). In our study, dronedarone produced a decrease in both SBP and HR, as described in previous clinical studies (Singh et al., 2007; Connolly et al., 2009; Quintana-Villamandos et al., 2017). By lowering the HR, dronedarone reduces oxygen consumption and increases diastole duration, thus improving coronary blood flow and, consequently, left ventricle performance (Heusch, 2008). Its antiadrenergic and calcium-antagonist properties are responsible for the reduction of SBP and could also contribute to coronary protection (Heusch et al., 2000).

### Effects of Dronedarone on p-NFATc4/ERK/AKT Pathway

Our study shows a negative regulation of the NFATc4 by dronedarone. NFATc4 represents a key component responsible for the induction of pathological hypertrophy (Bernardo et al., 2010; van Berlo et al., 2013; Tham et al., 2015). Previous preclinical studies have shown its effects upon inducing cardiac growth after pathological stimuli but not after physiological stimuli such as exercise (Bernardo et al., 2010; Carnegie and Burmeister, 2011; Schirone et al., 2017). Upon dephosphorylation by calcineurin, NFATc4 translocates to the nucleus and, by association with other transcription factors such as GATA4, induces the expression of genes linked to cell growth (Bernardo et al., 2010). NFATc4, which forms part of a larger family of transcription factors (NFATc1, NFATc2, NFATc3, NFATc4, and NFATc5), has demonstrated *in vivo* the ability to produce pathological LVH induced by isoproterenol (Li et al., 2011). This response would be prevented by NFATc4 downregulation through dronedarone as shown in the present study.

ERK1/2 is part of the larger mitogen-activated protein kinase (MAPK) family and has been linked to the development of cardiac hypertrophy (Daryadel et al., 2014). ERK1/2 is activated by numerous stimuli, such as endothelin, angiotensin II, or stress-induced hypertrophy (Bueno and Molkentin, 2002; Daryadel et al., 2014; Zhu et al., 2015; Ba et al., 2019). These activate G-protein coupled receptors that produce an activation cascade ending in the activation of MEK1/2, phosphorylation of ERK1/2 by MEK1/2, and induce its translocation to the nucleus and activation of numerous transcription factors such as GAT4A or p53 (Kehat and Molkentin, 2010). A previous study showed that p-ERK1/2 levels are significantly higher in neonatal SHR cardiomyocytes compared with neonatal WKY cardiomyocytes and that activation by endothelin was enhanced in SHRs and produced greater hypertrophy compared with WKY (Zhu et al., 2015), which is consistent with our results. However, ERK1/2 is not only related to cardiac hypertrophy but it seems to also have cardiac protective effects. Inhibition of this pathway has also produced an increase in apoptosis and thus, cardiomyocyte death (Harris et al., 2004; Lorenz et al., 2009; Kehat and Molkentin, 2010). In this line, Ruppert et al. (2013) described the phosphorylation of a tyrosine residue as an essential step to selectively induce pathological hypertrophy. Interestingly, they proved that interference with this phosphorylation did not affect physiological hypertrophy or anti-apoptotic properties.

AKT’s role in hypertrophy has been widely studied and is well known for its action upon stimulating cell growth and proliferation *via* activation by mTOR. AKT is also related to angiogenesis and vasorelaxation through activation of eNOS and stimulation of VEGF secretion (Abeyrathna and Su, 2015). The pathway represented by AKT has been commonly linked to physiological hypertrophy (Ersahin et al., 2015; Shimizu and Minamino, 2016). However, sustained activation of this pathway has proved to produce maladaptive hypertrophy and thus a negative effect on cardiac performance (Shimizu and Minamino, 2016; Schirone et al., 2017). This last statement is consistent with our study, which shows elevated levels of AKT in SHRs compared with normotensive controls and a decrease of those upon administration of dronedarone.

Signaling cascades are not independent of each other. Previous studies show how the activation of the calcineurin/NFAT pathway enhances ERK1/2 phosphorylation (Zou et al., 2001; Sanna et al., 2005) and the activation of ERK1/2 cascade increases p-NFATc4 activity (Sanna et al., 2005; Ramos-Kuri et al., 2015). Therefore, calcineurin-NFAT and MEK1-ERK1/2 signaling pathways are interdependent in cardiomyocytes where they directly coregulate the hypertrophic growth response. On the other hand, it is known that ERK1/2 and AKT pathways interact with each other by a negative regulation (Ersahin et al., 2015). However, our study and other previous experimental studies have also proved a positive regulation between them (Jiang et al., 2019; Saline et al., 2019). Interestingly, treatment with a multichannel blocker, such as dronedarone, decreases the levels of ERK, NFATc4, and AKT and, therefore, improves cellular signalization, after just 14 days of drug administration.

### Effects of Dronedarone on Thiol-Specific Oxidative Stress

Oxidative stress has been implicated in the development of cardiac hypertrophy (Maulik and Kumar, 2012). Reactive oxygen species are produced mainly in the mitochondria and may provoke an induction of various transcription factors responsible for pathological ventricular hypertrophy by enhancing the activity of signaling pathways such as ERK1/2 and NFATc4 (Maulik and Kumar, 2012; Sag et al., 2014; Moris et al., 2017).

The PTI is a new biomarker of oxidative stress that includes biomarkers of oxidative damage and antioxidant defense (Giustarini et al., 2012; Colombo et al., 2015). Recently, our group has shown that PTI could be a new biomarker of oxidative stress in patients with LVH: PTI was higher in patients with LVH compared with patients without LVH, the area under the receiver operating characteristic curve was 0.75, sensitivity was 70.6%, and specificity was 68.6%. Thus, PTI could be used to screen for LVH (Quintana-Villamandos et al., 2019). In the present study, we have observed the same result, the SHR control group showed a markedly higher PTI than the WKY control group. On the other hand, dronedarone improved the redox status by decreasing thiol-specific oxidative stress (PTI) in the same animal model of arterial hypertension and compensated LVH (SHR). Therefore, these results suggest that the decrease in PTI could be used as an indicator to monitor the efficacy of treatment with dronedarone if the results are confirmed in future clinical trials.

In conclusion, our study suggests that dronedarone produces inhibition of the NFATc4/ERK/AKT pathway and improvement of thiol-specific oxidative stress possibly secondary to the reduction of blood pressure in an animal model of ventricular hypertrophy.

## Limitations and Future Directions

Some limitations of our study should be mentioned. First, our study suggests that dronedarone decreases arterial pressure and produces an early reversal of LVH. Based on the presented data, one cannot conclude whether the effect of dronedarone is direct on cardiomyocytes or indirect. However, the literature indicates that systolic arterial pressure is the principal determinant of LVH regression in hypertensive humans (Verdecchia et al., 2004). Future studies in cell cultures could answer this question. Second, we only analyzed some components of the signaling pathways and oxidative stress. Considering the wide range of actions that this drug has, dronedarone could also have effects on other signaling pathways responsible for cardiac hypertrophy and could also exert other beneficial actions upon redox status. Finally, we must highlight as well that the present study is performed in a specific experimental model of arterial hypertension and compensated LVH. Thus, further studies will be necessary to determine the most suitable dose in humans to reproduce the positive effect of dronedarone in the LVH regression and to investigate if the mechanisms underlying it are also present.

## Data Availability Statement

All datasets presented in this study are included in the article/supplementary material.

## Ethics Statement

The animal study was reviewed and approved by Ethics Committee of Hospital General Universitario Gregorio Marañón, Madrid, Spain.

## Author Contributions

BQ-V and LP-S conceived the project and wrote the manuscript. BQ-V, MG, and LP-S performed the experiments, analyzed the data, and interpreted the results of experiments, and approved the submitted version.

## Conflict of Interest

The authors declare that the research was conducted in the absence of any commercial or financial relationships that could be construed as a potential conflict of interest.
